# Outcome and complications in horses administered sterile or non‐sterile fluids intravenously

**DOI:** 10.1111/jvim.15631

**Published:** 2019-10-14

**Authors:** Jamie J. Kopper, Megan E. Bolger, Clark J. Kogan, Harold C. Schott

**Affiliations:** ^1^ Department of Large Animal Clinical Sciences College of Veterinary Medicine, Michigan State University East Lansing Michigan; ^2^ Center for Interdisciplinary Statistical Education and Research, Washington State University Pullman Washington

**Keywords:** death, fluid therapy, morbidity, reverse osmosis water, thrombosis

## Abstract

**Background:**

Obtaining commercial fluids for intravenous administration (IVF) was challenging during a recent shortage. This necessitated use of custom‐made non‐sterile fluids for intravenous administration (JUGs) in some hospitals. There are no studies comparing outcome of horses treated with JUG versus IVF and limited information is available about adverse effects of JUGs.

**Hypothesis/Objectives:**

To evaluate death, complications, blood pH, and plasma electrolyte concentrations of horses that received JUG versus IVFs.

**Animals:**

One hundred eighty‐six horses that received IVFs and 37 that received JUGs.

**Methods:**

A retrospective review of medical records was performed to identify horses that received IVFs or JUGs during hospitalization. Information including survival to discharge, complications (fever [>38.5°C], jugular vein phlebitis/thrombosis, arrhythmia, or laminitis), blood pH, and plasma electrolyte concentrations were obtained.

**Results:**

There was no difference (*P* = .67) in survival to discharge for horses that received JUGs (78%) compared to horses that received IVFs (87%). Horses that received JUGs were more likely to develop a jugular vein complication (3 of 37 versus 1 of 186, odds ratio 17.2 [95% CI 1.9‐389.8], *P* = .04). Horses that received JUGs were more likely to have electrolyte abnormalities consistent with hyperchloremic metabolic acidosis.

**Conclusion and Clinical Importance:**

Veterinarians using JUGs should obtain informed client consent because of a potential increased risk of jugular vein complications. Chloride content of JUGs should be considered to limit development of hyperchloremic metabolic acidosis.

**Abbreviations**
AKI: acute kidney injury
CI: confidence interval
Cl^−^: chloride
ECF: extracellular fluid
ECG: electrocardiogram
HCO3^−^: bicarbonate
HR: heart rate
IV: intravenous
IVC: intravenous catheter
IVF: commercially obtained intravenous fluids for intravenous administration
JUG: non‐sterile fluids for intravenous administration
K^+^: potassium
LRS: lactated Ringer's solution
Na^+^: sodium
NSAID: nonsteroidal anti‐inflammatory drug
OR: odds ratio
Osm: osmolality
RR: respiratory rate
TS: total solids

## 1 INTRODUCTION

Fluids for intravenous (IV) administration are a crucial supportive treatment to restore and maintain vascular volume and tissue perfusion in horses with disease processes that can result in hypovolemia.^1^, ^2^, ^3^ Two examples potentially requiring fluids for IV administration include GI disease associated with large volume fluid loss (ie, diarrhea and gastric reflux) or instances when water consumption is precluded and enteral fluid therapy is not tolerated.

There are several limitations of IVF that are available in volumes practical for equine fluid therapy (ie, 3 and 5 L bags). First, most commercially available products are formulated as replacement solutions with electrolyte concentrations similar in composition to ECF. These products are appropriate for short‐term replacement of fluid deficits but might not be ideal for long‐term (>48 hours) supportive treatment of horses with ongoing fluid losses in gastric reflux or diarrhea, as they might provide an excessive load of sodium and chloride. Long‐term, high volume supportive treatment with replacement solutions will result in excess administration of Na^+^ and Cl^−^, promoting development of edema, hyperchloremic metabolic acidosis, and depletion of body K^+^ content through increased urine output.^1^, ^2^ Further, use of chloride‐rich IVF (ie, 0.9% NaCl) versus more balanced polyionic solutions (ie, Plasmalyte‐148) has been associated with increased morbidity and death in multiple studies in people.^4^, ^5^, ^6^, ^7^ Use of 0.9% NaCl in human patients results in the development of a hyperchloremic metabolic acidosis.^4^, ^6^, ^8^ The exact cause of increased death in human patients receiving 0.9% NaCl is not known. However, the use of 0.9% NaCl subsequently resulting in a hyperchloremic metabolic acidosis has been associated with decreased renal perfusion^9^ and impaired immune function^10^ including an increased risk of postoperative infections.^11^ Furthermore, in humans the use of replacement fluids in resuscitation of critically ill patients has specifically been associated with degradation of the endothelial glycocalyx further perpetuating poor vascular homeostasis.^12^

Moreover, the previous shortage of 3 and 5 L IVF bags for horses^13^ made obtaining IVF challenging at best, and impossible for some equine hospitals during that time period. This problem led to increased use of non‐sterile custom‐made fluids for IV administration formulated by adding bulk electrolyte products to reverse osmosis or distilled water carboys. A high rate of bacterial and low rate of endotoxin contamination in hospital prepared, non‐sterile fluids for IV administration was previously reported.^9^ However, potential adverse clinical signs associated with the administration of these fluids for IV administration were not reported in horses.

This retrospective study was performed to test the null hypothesis that horses treated with non‐sterile custom‐made fluids for IV administration (JUG) would not have increased rate of death or complications compared to horses treated with commercial, sterile IVF. Venous blood pH and plasma electrolyte concentrations of hospitalized horses were also evaluated to determine if horses receiving JUG would be more likely to develop acid‐base and electrolyte derangements, as compared to horses receiving IVF.

## 2 MATERIALS AND METHODS

### 2.1 Data collection

Medical records of horses greater than 6 months of age that were presented to Michigan State University's Veterinary Medical Center and received fluids administered IV between December 2014 and August 2017 were reviewed. Inclusion criteria included administration of IVF or JUG for at least 24 hours. Horses that were administered both IVF and JUG during the same hospitalization period were excluded. Data extracted from medical records included signalment, rectal temperature, HR, RR, PCV, TS, pH, lactate, and electrolyte (Na^+^, K^+^, Cl^−^, and HCO^3−^) concentrations at admission and ~24 hours after starting fluid therapy. The type(s) of fluids for IV administration administered and total volume (mL/kg) of fluids for IV administration administered were recorded. PCV and TS were determined by the microhematocrit method and refractometry, respectively, and the mean values of two samples recorded in medical records were used for analysis. Blood gas parameters, lactate and electrolyte concentrations were determined with a blood gas analyzer (Novaphox Ultra, Novabiomedical, Waltham, Massachusetts). Outcome (survival to discharge) and development of a fever (rectal temperature >38.5°C), jugular vein phlebitis/thrombosis, arrhythmias, and laminitis were the endpoints of interest.

All IVCs used for horses in this study were 14 gauge × 9 cm polyurethane catheter (Mila International, Inc, Florence, Kentucky) that were inserted by a member of the veterinary care team (veterinarian or licensed veterinary technician). A standard protocol of aseptic skin preparation, injection of 1.0 to 1.5 mL of a 2% lidocaine HCl solution SQ over the jugular vein, and retention of catheters with suture was followed during the study. Although extension sets attached to the catheter hub varied during the course of the study (not documented in medical records), neck bandages to cover the catheter insertion site were not used and catheter insertion sites were only cleaned if blood had accumulated at the insertion site. IVCs and the associated jugular vein were evaluated for complications (swelling, heat, pain or discharge as well as thrombosis) four times daily during routine physical examinations and replaced if deemed necessary by the attending veterinarian.

To assess for complications, all pages of the medical records were carefully reviewed for documentation of a fever, jugular vein phlebitis/thrombosis, arrhythmias, and evidence of laminitis (weight shifting, increased digital pulses, sensitivity to hoof testers at the apex of the frog). Jugular vein phlebitis was defined as palpation of vessel wall thickening (with heat, pain, or both) on routine examination and jugular vein thrombosis was defined as complete occlusion of the vessel confirmed by absence of jugular vein distention when the vein was occluded at the base of the neck, below the catheter insertion site.

#### 2.1.1 Fluids for intravenous administration

If fluids for IV administration were needed for supportive care of horses during the study period, owners were offered either IVF or JUG fluids. Owners were informed of the cost difference and the projected impact on the financial estimate provided as well as provided with the perceived risks of using JUG fluids. Given that there were not current studies evaluating the effect of JUG fluids on outcome in horses owners were informed of our perceived risks at that time which included the presence of bacteria, endotoxin or both within the fluids which could result in an increased risk of complications and decreased survival to discharge. Owners were allowed to choose which fluid type they preferred for their horse to receive and could change their mind at any time during hospitalization. IVF used during the study period included Lactated Ringer's solution (Hospira, Lake Forest, Illinois), Vetivex solution (Dechra Veterinary Products, Overland Park, Kansas) and Plasma‐Lyte 148 Replacement solution (Baxter International, Deerfield, Illinois). Three custom‐made JUG formulations were also used during the study period and were selected at the attending clinician's discretion based on patient information (primarily serum electrolyte, acid base status, projected continued losses, and creatinine concentration). Composition and tonicity of IVF and JUG are detailed in Table 1.

**Table 1:** Composition of commercial fluids and custom‐made, non‐sterile fluids for IV administration (JUG) used during the study period. All solutes are expressed as mmol/L and osmolarity is expressed as mOsm/L

Fluid type	Na^+^	Cl^−^	K^+^	Ca^++^	Mg^++^	Lactate	Acetate	Gluconate	Osm
LRS	130	109	4	2.7	‐	28	‐	‐	273
Vetivex	131	111	4	3	‐	29	‐	‐	278
Plasma‐Lyte 148	140	98	5	‐	‐	‐	27	23	295
JUG 0.9% NaCl	153	153	‐	‐	‐	‐	‐	‐	306
JUG Rehydration	122	152	30	‐	‐	‐	‐	‐	306
JUG Maintenance	92	152	59	‐	‐	‐	‐	‐	303

### 2.2 Data analysis

Horses were grouped by type of IV fluid administered (IVF or JUG) and missing data was accounted for by imputation of the mean. A propensity score analysis was conducted to estimate the effect of the type of IV fluid administered on survival to discharge and development of fever, jugular vein phlebitis/thrombosis, arrhythmia, and laminitis. The propensity score was used in a regression adjustment in order to account for risk factors that were potential confounders of the outcome variables, while avoiding the degree of overfitting that could result from directly adjusting for all confounders in the model.^14^ The score was constructed through a logistic regression analysis that included the following factors: patient age, admission HR, RR, PCV, TS, lactate concentration, and total mL/kg of fluids administered during hospitalization. The effect of receiving JUG on the aforementioned binary outcome variables was assessed with a logistic regression that included the log odds of the propensity to be given JUG, the treatment group (IVF or JUG), and two primary risk factors: admission HR and admission lactate concentration. The effect of receiving JUG on blood pH and plasma Na^+^, Cl^−^, K^+^, and HCO^3−^ concentrations ~24 hours after initiating IV fluid therapy were assessed via linear regression where the treatment group and admission electrolyte values were included as independent variables. Post hoc power calculations for detection a difference in outcomes that did not reach statistical significance were performed using an alpha level of .05 and a power of .80. All statistical analyses were performed by R (R Core Team 2017) with significance set at *P* < .05.

## 3 RESULTS

### 3.1 Horses and treatments

Two hundred and ninety‐nine horses were treated with fluids for IV administration during the 2.5 year study period. Seventy‐six horses were excluded because they received both IVF and JUG during the same hospitalization, the owners elected euthanasia in surgery or they had incomplete medical records. One hundred eighty‐six horses that received IVF and 37 horses that received JUG were included in the final analyses. Mean age (IVF 12.8 and JUG 13 years) and sex distribution were similar between the two groups and a variety of breeds were represented.

### 3.2 Admission data

Admission physical exam and clinicopathologic data are detailed in Table 2. Admission parameters were not significantly different between the two groups.

**Table 2:** Median values (25, 75% interquartile range) for selected patient data for horses that received commercial fluids for IV administration (IVF) and custom‐made, non‐sterile fluids for IV administration (JUG) during the study period. The number of horses for which these values were available varied and are included in each box

	Age (years)	HR	RR	PCV (%)	TS (g/dL)	Lactate (mmol/L)	mL/kg fluid administered	Hours with IVC
IVF	14	51	24	39	6.7	1.4	90	75
	(8,18)	(44,60)	(18,32)	(35,45)	(6.2,7.5)	(0.9,3)	(60,150)	(55,187)
	n = 186	n = 186	n = 184	n = 181	n = 181	n = 172	n = 186	n = 186
JUG	13	52	24	40	6.9	1.5	80	69
	(9,20)	(48,64)	(18,36)	(36,44)	(6.2,7.7)	(1.1,2.4)	(50,120)	(52,149)
	n = 37	n = 37	n = 36	n = 35	n = 34	n = 33	n = 37	n = 37

### 3.3 Survival and complications

There was no difference in survival between horses receiving IVF (87%) and JUG (78%) (*P* = .67). However, development of jugular vein phlebitis/thrombosis was more likely (*P* = .01) in horses that received JUG (3/37) than in horses that received IVF (1/186) (OR 17.1, 95% CI: 2.0‐389.9). There were no differences between IVF and JUG in development of fever (*P* = .08, OR 1.99 95% CI: .927‐4.19), arrhythmia (*P* = .51, OR 8.34 × 10^−8^ with an unbounded 95% CI) or laminitis (*P* = .09, OR 1.90, 95% CI: .71‐71.81) (Table 3). Ventricular tachycardia developed in two horses, both in the IVF treatment group.

**Table 3:** Survival to discharge and morbidity of horses that received commercial fluids for IV administration (IVF) and custom‐made, non‐sterile fluids for IV administration (JUG) during the study period

	Survival to discharge	Jugular vein complication	Fever at 24 hours	Arrhythmia	Laminitis
IVF	87%	.5%	24%	1.0%	1.0%
	162/186	1/186	44/186	2/186	2/186
JUG	78%	8.1%	35%	0%	5.4%
	29/37	3/37	13/37	0/37	2/37
*P*‐value	.67	**.01**	.08	.51	.09
Odds ratio (95% confidence interval)		17.2	2.0	8.3 × 10^−8^	6.7
		(1.9,389.8)	(0.9,4.2)	a	(0.7,71.8)

*Note*: Bolded values are indicative of a *P* value of less than 0.05.

a Confidence intervals are unbounded.

### 3.4 Changes in clinicopathologic values

For horses with serial venous blood gas analyses, treatment with JUG resulted in a lower blood pH (7.37 versus 7.42, *P* = .02) and higher Cl^−^ concentration (107 mmol/L versus 106 mmol/L, *P* = .03) after ~24 hours compared to horses that received IVF. Horses receiving JUG fluids also had a lower HCO3^−^ concentration compared to horses receiving IVF (18.4 mmol/L versus 22.4 mmol/L, *P* = .005) after 24 hours (Table 4).

**Table 4:** Values (median [25% and 75% interquartile ranges]) for venous blood pH and plasma electrolyte concentrations (mmol/L) at admission and after ~24 hours of IV fluid therapy) for horses that received commercial fluids for IV administration (IVF) and custom‐made, non‐sterile fluids for IV administration (JUG) during the study period. The number of horses (n) for which these values were available is noted in each box

Admission	pH	HCO_3_^−^	Na^+^	K^+^	Cl^−^
Reference Interval	7.38‐7.42	22.7‐31.2	135‐140	2.1‐4.0	103‐110
IVF	7.42 (7.39,7.44) n = 172	24 (21.5,26.4) n = 171	136 (134,138) n = 176	3.5 (3.2,3.8) n = 175	104 (102,106) n = 175
JUG	7.43 (7.4,7.45) n = 33	25 (20.5,27.4) n = 33	134 (132,137) n = 34	3.4 (3.0,3.9) n = 34	102 (99,105) n = 33
24 hours	pH_24h_	HCO_3_^−^_24h_	Na^+^_24h_	K^+^_24h_	Cl^−^_24h_
IVF	7.42 (7.36,7.44) n = 35	22.2 (20.9,24.0) n = 35	136 (134,139) n = 36	3.4 (2.9,3.7) n = 36	106 (104,108) n = 36
JUG	7.37 (7.34,7.38) n = 5	18.4 (18.4,19.1) n = 5	134 (128,136) n = 5	3.9 (3.8,4.6) n = 5	107 (105,108) n = 5
*P* value	**.015**	**.005**	.55	.81	**.034**

*Note*: Bolded values are indicative of a *P* value of less than 0.05.

## 4 DISCUSSION

An increased risk of death was not found in horses that received JUG fluids in this study. Further, use of JUG fluids did not significantly increase the risk of development of a fever, an arrhythmia or laminitis. However, use of JUG fluids was associated with increased risk of jugular vein complications (specifically thrombosis or thrombophlebitis), as well as development of a mild hyperchloremic metabolic acidosis, when compared to horses that received IVF.

Jugular vein phlebitis/thrombosis is a well‐recognized complication with use of IVCs.^15^, ^16^, ^17^, ^18^, ^19^, ^20^ The classic triad, as described by Virchow in 1856, leading to intravascular thrombosis includes blood vessel trauma, stasis of blood flow, and a hypercoagulable state.^15^, ^21^, ^22^, ^23^ Risk factors for jugular vein phlebitis/thrombosis include IVC composition, venipuncture technique, length and diameter of the catheter, catheter maintenance protocols, pH of IV solutions, duration of catheter placement, and sepsis associated with the catheter site.^17^, ^18^, ^19^, ^20^, ^24^, ^25^, ^26^ Despite having a consistent procedure in place for placement of IVCs during the study period, there might have been variation between veterinarians/technicians, as well as horse behavior, during IV catheter placement that might have affected risk of developing phlebitis/thrombosis. Further, coagulation status, a documented risk factor,^15^ was not routinely assessed in horses in this study. Although horses receiving JUG fluids were 17 times more likely to develop a jugular phlebitis/thrombosis, supporting fluid type as a risk factor for this complication, it warrants emphasis that absolute numbers of jugular vein complications documented in medical records were low with both types of fluids. For comparison, in another study jugular vein thrombosis was found in 20 of 69 (29%) horses that received fluids for IV administration for >24 hours and presence of fever or diarrhea, along with use of custom‐made fluids, were significant risk factors.^19^ Similarly, jugular vein thrombophlebitis was a short‐term complication in 21/252 (7.5%) horses after colic surgery, with the incidence higher in horses manifesting postoperative pain or shock (15 and 20%, respectively).^27^ Realistically, reliance on information retrieved from medical records likely resulted in underestimation of jugular vein complications in our study. As an example, when more critical examination was pursued in a study comparing two catheter types, 61% of veins had local perivascular swelling, hematomas or both and ultrasonographic assessment revealed moderate to severe venous pathology in 5.4% of catheterized veins.^16^ Although JUG fluids likely had a greater risk of contamination with bacteria and endotoxin, neither of which are found in commercial fluids,^28^ introduction of these agents would more likely produce systemic effects (eg, fever, tachycardia, tachypnea, and sweating)^29^ rather than localized inflammation or sepsis. Consequently, the mechanism(s) by which custom‐made, non‐sterile fluids might have contributed to an increased risk of jugular vein complications was not fully elucidated in either our or previous studies.^19^

To the best of our knowledge, none of the horses in this study suffered further complications of jugular vein phlebitis/thrombosis after hospital discharge. However, sequela can include ipsilateral swelling of the head and neck, bacteremia, endotoxemia, pulmonary infarction, and vegetative endocarditis.^25^, ^30^ Further, thrombosis resulting in permanent occlusion of a jugular vein would make future medical treatment requiring placement of an IVC into the contralateral jugular vein of greater risk for more severe complications, including head swelling and airway occlusion. Unilateral jugular vein thrombosis has also been hypothesized to limit athletic potential; however, a retrospective study found no significant difference in performance of either pleasure or racehorses before and after development of a jugular vein thrombosis, in horses that returned to performance.^31^

A guiding principle of fluid therapy is restoration and maintenance of euvolemia and tissue perfusion, while avoiding exacerbation of metabolic disturbances. In humans, use of IVF containing Cl^−^ in concentrations higher than patient serum (or “chloride rich fluids”) promotes development of hyperchloremic metabolic acidosis and increased morbidity and risk of death with this metabolic disturbance has gained attention in recent years.^4^, ^11^, ^32^, ^33^ In people, development of hyperchloremia has been associated with decreased renal blood flow and renal cortical perfusion,^9^ impaired immune function,^10^ increased postoperative infection rates^11^ and increased risk of death.^11^, ^34^, ^35^ Further, in people restriction of Cl^−^ administration in IVF has been associated with lesser increases in creatinine during hospitalization, indicating protection against AKI, when compared to when IVF higher in [Cl^−^] were used.^4^ Unfortunately, at this time, the veterinary literature is lacking studies assessing the potential effects of hyperchloremia on patient outcome. The JUG formulas used during the study period had higher [Cl^−^] than those found in the IVF, and use of JUG fluids was found to be associated with development of hyperchloremic metabolic acidosis. After this potential problem was identified, the JUG formulas used in our hospital were modified to provide less Cl^−^ by substituting some KCl with KHCO^3−^. The potential impact of this change has not yet been assessed.

There are several limitations of this study that should be acknowledged. First, the number of horses in each group was small and unbalanced and 24 hours clinicopathologic parameters were measured in a limited number of patients, including only five horses receiving JUG fluids. Incomplete data has the potential to introduce bias (ie, patients that were not responding as well were more likely to have blood work repeated). Further, IVF and JUG treatments were heterogeneous, with both IVFs and JUGs with different electrolyte compositions administered to horses in the study. It would have been ideal to categorize horse groups by brand of IVF and JUG formula; however, this would have resulted in even smaller group sizes. Given that the primary research question was the effect of sterile and non‐sterile fluids for IV administration on outcome variables, rather than the impact of electrolyte composition, we chose to group horses based on administration of IVF or JUG. A prospective multicenter clinical trial comparing a single IVF and a single custom‐made fluid, with similar ionic compositions, and standardized reassessments would provide the best quality evidence to further address our hypothesis. Second, it would have been valuable to know if the JUG fluids used in this study were contaminated with bacteria, endotoxin or both. Based on a prior study^22^ it is likely that JUG fluids were contaminated. Moreover, it would have been interesting to know whether or not development of jugular vein complications was associated with the magnitude of contamination. Further, because pH of IVF has been documented as a risk factor for developing phlebitis and thromboses,^24^ it would have been useful to have measured pH of JUG formulas. Third, as mentioned medical records likely underreported development of jugular vein phlebitis/thrombosis, based on rates of thrombophlebitis documented by more critical assessment in previous studies.^16^, ^19^, ^27^ In addition, it is possible that jugular vein complications developed after discharge from the hospital. Because owners were not contacted for long‐term follow‐up, it is possible that this complication was missed in some horses. Fourth, continuous ECG monitoring was not performed unless an arrhythmia was detected by auscultation. Thus, it is possible that transient arrhythmias were missed with intermittent (every 6 hours) auscultation. Fifth, administration of NSAIDs might have limited fever development, both at admission and after ~24 hours. Treatment with NSAIDs was not assessed as a risk factor in the statistical analysis because of a number of factors including: variable NSAID administration before presentation, differences in clinician preference regarding administration of NSAIDs (timing and dose) and the fact that essentially all horses received 1 or more doses of an NSAID during the initial 24 hours of hospitalization. It should also be noted that although a statistical significance was not reached for fluid type and proportion of horses that developed laminitis or a fever within 24 hours the *P*‐values were <.10, which might be cause for consideration. ORs and 95% CIs were provided so that the reader can determine their level of comfort with these results. Post hoc power calculations indicated that to detect a difference in the proportion of horses affected by laminitis a total of 344 horses per group would have been required and a total of 176 horses per group would have been required to detect a difference in the proportion of horses that had a fever recorded in the first 24 hours after starting fluids. Finally, given the significant difference in cost between the two types of fluids administered, it is possible that financial constraints of the owner were an additional confounding factor.

In conclusion, we found no evidence that horses receiving JUG fluids were less likely to survive to discharge compared to horses that received IVF. However, there was moderate evidence that horses that received JUG fluids had an increased risk of developing a jugular vein complication. When considering use of JUG fluids, this increased risk should be discussed with owners so that they can make an informed decision. Additionally, use of Cl^−^‐rich JUG formulas resulted in hyperchloremic metabolic acidosis compared to those horses that received IVF. Consequently, hospitals using custom‐made, non‐sterile fluids for IV administration should consider the amount of Cl^−^ when formulating fluids for IV administration.

## CONFLICT OF INTEREST DECLARATION

Authors declare no conflict of interest.

## OFF‐LABEL ANTIMICROBIAL DECLARATION

Authors declare no off‐label use of antimicrobials.

## INSTITUTIONAL ANIMAL CARE AND USE COMMITTEE (IACUC) OR OTHER APPROVAL DECLARATION

Authors declare no IACUC or other approval was needed.

## HUMAN ETHICS APPROVAL DECLARATION

Authors declare human ethics approval was not needed for this study.
